# Modeling of Fracture Toughness of Degraded HR3C Steel in Relation to Microstructural Changes

**DOI:** 10.3390/ma19081581

**Published:** 2026-04-15

**Authors:** Jakub Horváth

**Affiliations:** Department of Materials Engineering, Faculty of Mechanical Engineering, Czech Technical University in Prague, Charles Square 13, 12135 Prague, Czech Republic; jakub.horvath@cvut.cz; Tel.: +420-224-357-249

**Keywords:** austenitic steel, HR3C, fracture toughness, precipitation changes, M_23_C_6_, ultra super critical powerplants (USC)

## Abstract

**Highlights:**

The article documents the change in fracture toughness of HR3C steel in relation to its thermal degradation. It considers the influence of precipitates formed at grain boundaries, which leads to a sharp drop in the mechanical properties of the steel. In relation to LMP, it graphically shows the decrease in fracture toughness and thus the material’s resistance to crack propagation. This has a direct basis for use in modeling the behavior of a superheater with cracks formed, for example, from operation, degradation or as an effect of welding errors.

**What are the main findings?**

- The main findings are a description of the dependence of fracture toughness of HR3C to thermal exposition through direct, quantitative correlation between the length fraction of grain boundaries occupied by M_23_C_6_ precipitates and the drop in fracture toughness of HR3C steel. This description provides an applicable methodology for the degradation description of operated thermal exchangers.

**What are the implications of the main findings?**

- Ability to calculate the critical size of cracks.
- FEM toughness analysis of degraded HR3C steel.
- Refinement of residual life calculation.

**Abstract:**

The article documents the cause of a sharp decrease in the fracture toughness of HR3C austenitic steel intended for heat exchange surfaces of supercritical energy blocks during its exposure to elevated temperature. The documentation of the cause of the decrease in fracture toughness is based on a combination of fractographic observation of the fracture surfaces of the tested samples, linked through ongoing precipitation changes in the steel to the fracture toughness of the steel. The result is a description of the decrease in fracture toughness in relation to the Larson–Miller parameter and subsequently the change in fracture toughness in relation to the precipitation changes in HR3C steel. This dependence provides a tool for numerical calculations and simulations of heat exchange surfaces of power plants made of HR3C steel and the simulation of their behavior when cracks are present.

## 1. Introduction

Austenitic corrosion-resistant, creep-resistant steel HR3C is one of the few materials intended primarily for heat exchange surfaces of supercritical or ultra-supercritical power plants. This is a material that should guarantee high creep resistance and at the same time satisfactory corrosion resistance to both flue gas and steam oxidation on the inside surfaces of the heat exchanges. However, the target application of HR3C steel involves exposure to a combination of elevated pressures and high temperatures, which will accelerate degradation. The degradation of the material will affect the mechanical properties and creep resistance of the steel.

The literature provides documentation of changes in basic mechanical properties such as tensile strength or impact strength. The literature [1,2] documents microstructure changes in the point of view of precipitation of chromium carbides at the grain boundaries. In the literature [3], the authors deal with HR3C steel mainly from the point of view of creep testing. The precipitation at the grain boundaries is identified here, which, like the previous authors, confirms the precipitation of M_23_C_6_ carbides at the grain boundaries during temperature exposure of HR3C steel. A clear description of precipitation with increasing exposure in HR3C steel is published in the work [4].

In general, attention is paid to HR3C steel in other articles, mainly from the point of view of creep, which is understandable given the targeted application of the material for heat exchange surfaces. In the literature, it is also possible to find descriptions of significant changes in mechanical properties that generally document the embrittlement of HR3C steel during its temperature exposure [5,6,7]. An example is the article [5] in which the samples were evaluated after 1000 and 5000 h of heat exposure from the point of view of the decrease in impact strength.

For HR3C steel, the precipitation mechanism is well described in the literature, primarily involving M_23_C_6_ carbides. Among others, the description of precipitation is dealt with in articles [8,9,10]. Unfortunately, in order to assess the degraded state of HR3C steel, none of the articles pay attention to fracture toughness. The article [11] documents the principal embrittlement for HR3C steel in the state before thermal exposure. However, its conclusions are not valid for the thermally exposed state of the material. Precipitation is generally problematic for materials intended for energy applications. Results for welded joints of SUPER 304H steel are presented in [12]. The article documents the decrease in mechanical properties of welded joints and points to future application problems.

Unfortunately, those data are insufficient for numerical simulations of the load on heat exchange surfaces with cracks in operation. The problem is how to deal with the heat exchange surface after performing diagnostics that identify cracks and are able to decide whether the defects present are critical or not.

The fact is that cracks are present on the real components of the USC power plants, and none of the articles address what change will occur from the point of view of the fracture toughness of the material, i.e., a change in its resistance to crack propagation, which can have more fatal consequences for the operation of HR3C steel than creep deformation.

The main problem with the literature data available for HR3C material is that fracture toughness data for the degraded state of HR3C steel are not available. This article attempts to fill this gap and provide data for the degraded state of HR3C steel.

The main objective of this article is to document and describe the relationship between the change in microstructure caused by thermal degradation of HR3C steel and the change in fracture toughness, so as to create a tool for determining fracture toughness in practice without the need for destructive intervention in the pressure system of a power plant.

## 2. Materials and Methods

### 2.1. Experimental Material

For the measurements and tests in this article, steel HR3C with a chemical composition shown in Table 1 was used. The dimensions of the tubes used for the preparation of the test material were 38 mm × 6.3 mm. The heat treatment of the base material was carried out at 1250 °C for two minutes and then cooled with a water shower.

For the needs of the article, it was necessary to use accelerated thermal degradation, which simulates the operational life of HR3C steel. The samples were exposed via isothermal annealing in laboratory furnaces at an elevated temperature of 675 °C for approximately 20, 50, 100, 500 and 2400 h. The samples were divided into groups, which were marked as follows: R0 for the supplied base material, R1 for an exposure load of 20 h/675 °C, R2 50 h/675 °C, R3 100 h/675 °C, R4 500 h/675 °C, and the last group with the highest state of degradation, R5 2400 h/675 °C. This is a set of samples that should cover, when considering accelerated degradation, up to 9 × 10^4^ h of conventional operation of the power unit for an operating steam temperature of 610 °C. This is a comprehensive set of samples that includes equivalent material degradation corresponding to the entire life cycle of a power plant unit.

In practice, parametric equations such as the Larson–Miller parameter are commonly used to evaluate degradation and creep state, which combine the effects of temperature and time. The main principle of applying these parametric equations is based on the fact that the degradation process that causes changes is the same during exposure. This principle is also used in this article because the controlling process of fracture toughness change was identified as carbide precipitation, which is a thermally activated process and also corresponds to material degradation under normal operating conditions.

For better clarity and subsequent application of the results to practice, equivalent values of the Larson–Miller parameter (LMP) were used for evaluation, which will allow generalizing the results according to the operating temperature of the power plant.

### 2.2. Evaluation of Carbide Precipitate Content Along Grain Boundaries

A combination of scanning electron microscopy (JEOL JSM-7600F scanning electron microscope, JEOL, Tokyo, Japan) and image analysis of micrographs (Nis Elements AR ver 4.1 software) was used to determine boundary occupancy by precipitates. Image analysis was based on manual detection of precipitates at grain boundaries and determination of their length in the direction of the grain boundary. Subsequently, the length of the grain boundary was measured on the micrograph, to which the degree of occupancy by precipitates was related. For each group of tested samples, 30 micrographs were used. With an average grain length of 85 mm/mm^2^, the evaluated grain boundary length corresponds to 688 mm of the total grain boundary length for a single sample group.

Based on the fact that particles precipitated at grain boundaries grow preferentially in the direction of grain boundary length, the value of grain boundary occupation length (BOL) by precipitates was used to describe the amount of precipitation.

### 2.3. Determination of Fracture Toughness

To determine the fracture toughness of the initial and thermally exposed state of HR3C steel, the method of three-point bending of test specimens with a projected crack was used. The evaluation of the tests was carried out according to the ASTM E1820-11 [13] Measurement of fracture toughness.

Fracture toughness measurements were performed for each group (R0 to R5) on a set of at least 4 samples. The initial set of samples always contained 5 test specimens; unfortunately in some cases, in groups R1, R4 and R5, one specimen was unevaluable. Therefore, it is stated that at least 4 specimens were used for evaluation. In the case of group R4, the raw data record for one specimen was also damaged; for this reason, only three specimens were evaluated, although consistency was evident between all specimens, including the damaged one, during testing, namely, reduced bodies for three-point bending with a body thickness of 3 mm. Unfortunately, the size of the semi-finished products (tubes) did not allow for larger testing bodies. Although miniaturized specimens are limited in terms of stress distribution at the crack tip and thus difficult to generalize to thick-walled components, the methodology is based on the fact that the dimensions of heat exchanger tubes are generally manufactured in only two wall thicknesses that correspond to the tested specimens, so the fracture toughness values will be applicable in this area. Cracks were pre-prepared in the samples by high-frequency cycling and were subsequently subjected to an instrumented dynamic test. This is therefore a determination of dynamic fracture toughness on reduced bodies. The description of individual quantities is given in the list of abbreviations.

The following calculations were used:

(1) $$J=\frac{{K_{i}}^{2}\left(1-v^{2}\right)}{E}+\frac{\frac{{1.9A}_{pl}}{B_{N}b_{0}}}{1+\left(\frac{\alpha -0.5}{\alpha +0.5}\right)\frac{∆a}{b_{0}}}$$

(2) $$K_{i}=\left[\frac{F_{i}S}{{\left(BB_{N}\right)}^{1/2}W^{3/2}}\right]f\left(\frac{a_{i}}{W}\right)$$

(3) $$f\left(\frac{a_{i}}{W}\right)=\frac{3{\left(\frac{a_{i}}{W}\right)}^{1/2}\left[1.99-\left(\frac{a_{i}}{W}\right)\left(1-\frac{a_{i}}{W}\right)\left(2.15-3.93\left(\frac{a_{i}}{W}\right)+2.7{\left(\frac{a_{i}}{W}\right)}^{2}\right)\right]}{2\left(1+2\frac{a_{i}}{W}\right){\left(1-\frac{a_{i}}{W}\right)}^{3/2}}$$

(4) $$K_{JC}=\sqrt{\frac{JE}{\left(1-v^{2}\right)}}$$

## 3. Results

The experimental part is divided into three subsequent sections: fractography analysis of fracture surfaces, evaluation of microstructural changes and their description, and the last determination of fracture toughness for groups of samples.

### 3.1. Change in Fracture Morphology Due to Temperature Exposure

**Figure 1:**
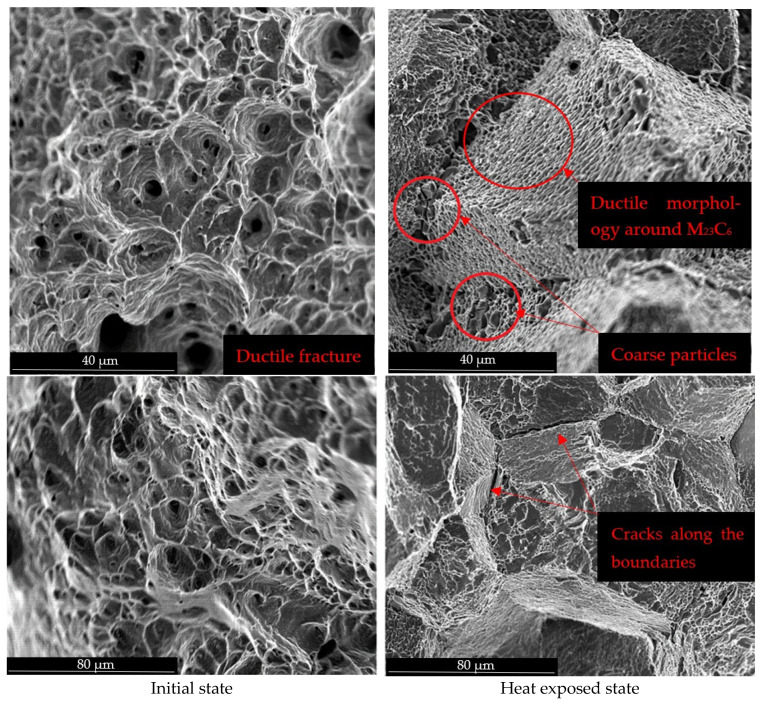
Fracture surfaces of steel HR3C.

The failure mechanism, either statically or dynamically, can be evaluated for HR3C austenitic steel in its initial state before thermal degradation as a ductile failure with a characteristic pit morphology. The failure development can also be evaluated as transcrystalline (left column in Figure 1). On the other hand, a very short temperature exposure leads to precipitation at the grain boundaries, which results in a change in the fracture development of the degraded HR3C steel in the form of crack development along the grain boundaries, i.e., intercrystalline. Furthermore, it is possible to observe that M_23_C_6_ precipitates at the grain boundaries act as an accelerating element of crack propagation. In general, it is possible to evaluate carbides as brittle particles, which corresponds to the deformation of the matrix just around the brittle fractured carbides. As the exposure time increases, the amount of carbides at the grain boundaries increases, which should logically lead to a further weakening of grain boundary cohesion and, in this case, to a decrease in dynamic mechanical properties as well as resistance to crack propagation along grain boundaries (right column of Figure 1).

### 3.2. Boundary Occupancy Length

Based on the described changes in the fracture development of HR3C steel with its thermal degradation, the precipitation evaluation was focused on the description of the increase in the occupancy of grain boundaries by M_23_C_6_ carbides.

**Figure 2:**
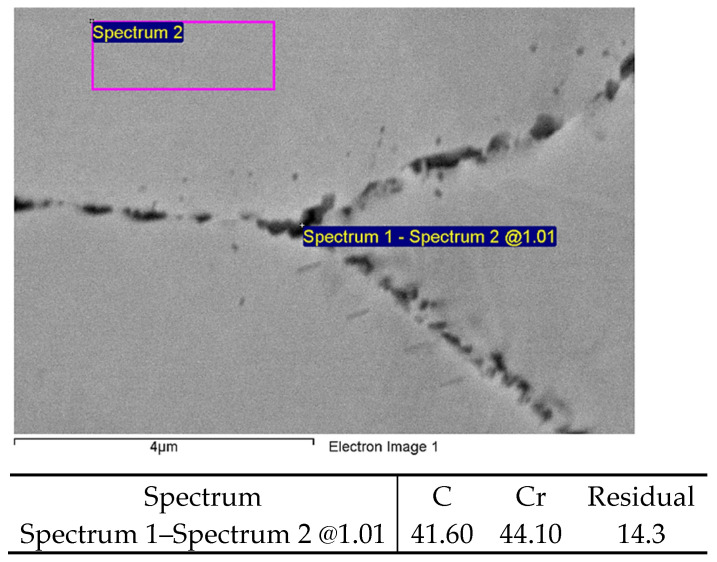
Verification of M_23_C_6_ carbide precipitation at the grain boundary (wt.%).

As a first step, the verification of the fact that, chemically, it really is M_23_C_6_ carbide with a dominant representation of M in the form of chromium was carried out using EDS analysis. Due to the size, it was necessary to perform a measurement in the form of the difference in intensities between the spectrum of the matrix and the spectrum that affects the particle and the steel matrix at the same time. The results are shown in Figure 2 and confirm that the grain boundary precipitates are chromium-based carbides.

**Figure 3:**
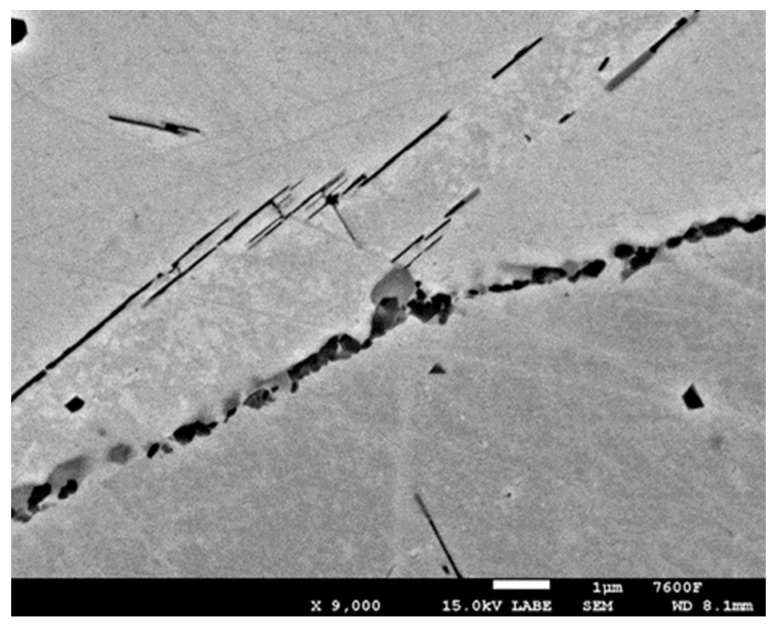
Detail of M_23_C_6_ precipitation along grain boundary.

In the detailed micrograph of the grain boundary (Figure 3), it is possible to observe that the precipitation of chromium carbides occurs discontinuously, the primary growth of carbides is along the grain boundary, and the individual carbides are gradually joined into a continuous envelope along the grain boundary. Thanks to this, it is possible to measure the percentage occupancy of grain boundaries, as described in the Section 2.

**Figure 4:**
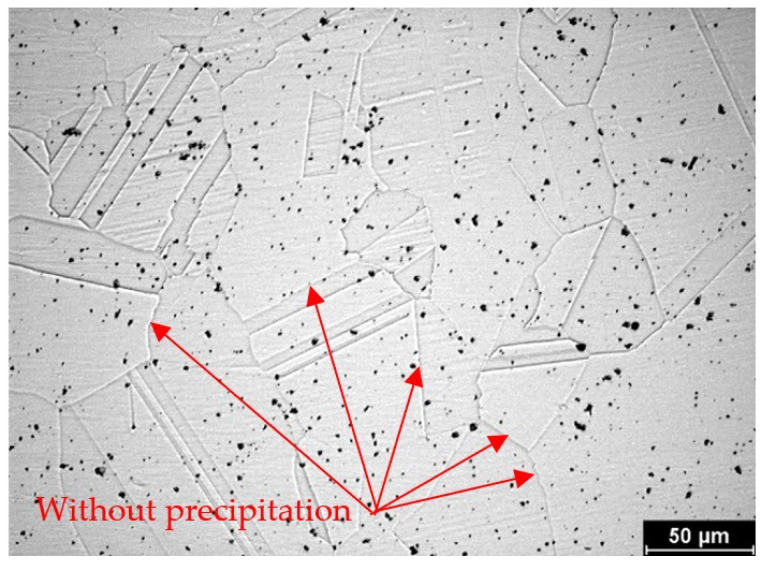
Microstructure of HR3C steel from group R0.

**Figure 5:**
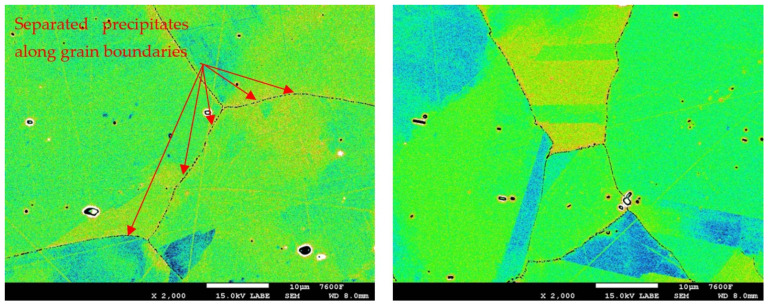
Images used for the grain boundary occupancy for sample group R1.

**Figure 6:**
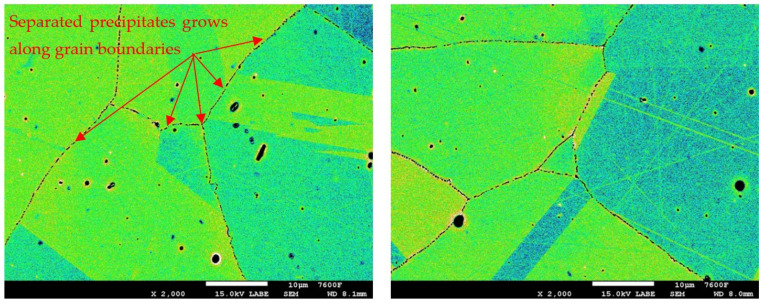
Images used for the grain boundary occupancy for sample group R2.

**Figure 7:**
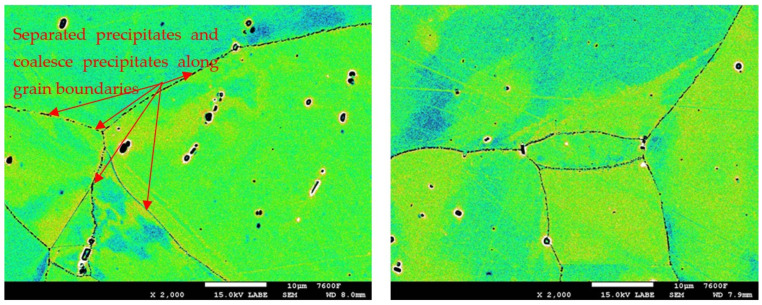
Images used for the grain boundary occupancy for sample group R3.

**Figure 8:**
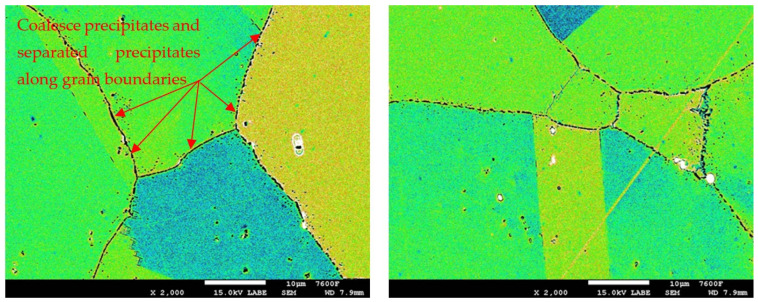
Images used for the grain boundary occupancy for sample group R4.

**Figure 9:**
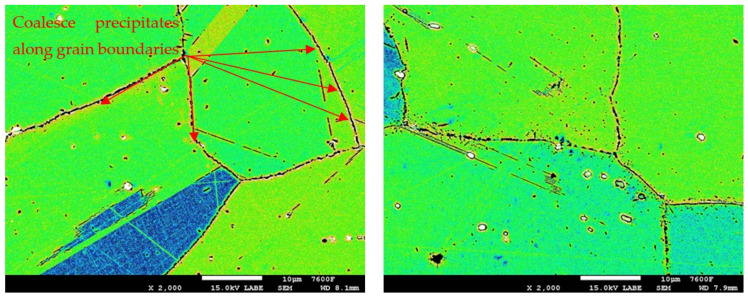
Images used for the grain boundary occupancy for sample group R5.

In the case of the group of samples R0 (Figure 4), which correspond to the supplied base material, no precipitates of chromium carbides were observed at the grain boundaries. From the comparison of characteristic images for groups of samples R1 to R5 documenting the precipitation of chromium carbides at grain boundaries with increasing time of temperature exposure, it is possible to observe a gradual increase in the occupancy of grain boundaries (Figure 5, Figure 6, Figure 7, Figure 8 and Figure 9).

**Figure 10:**
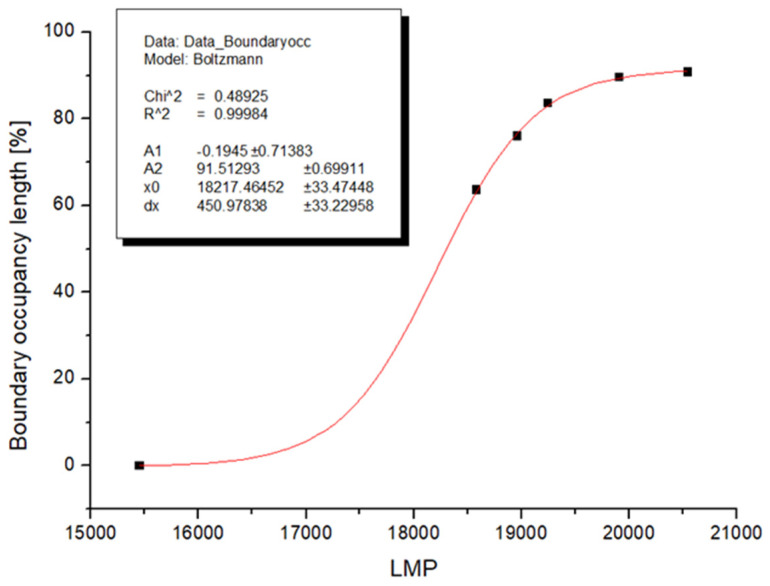
Boundary occupancy length to LMP parameter for steel HR3C.

Based on the measurement of the occupancy of grain boundaries by chromium carbides, the following dependence on LMP was plotted. The conventional equation LMP = T(C + log(t)) was used to calculate the LMP parameter. The constant C was taken from creep tests for HR3C steel, namely C = 18.3. Fitting by sigmoidal functions, which are generally suitable for describing transition events, achieved a function determination coefficient of 99.98%, which proves that the description of chromium carbide precipitation events with this function is very accurate. Functional coefficients are summarized in the graph (Figure 10).

### 3.3. The Fracture Toughness Measurement

**Figure 11:**
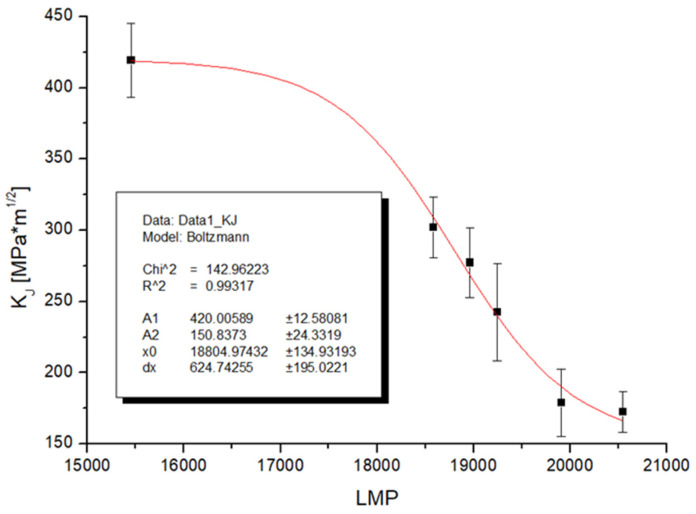
Fracture toughness to the LMP parameter for steel HR3C.

Fracture toughness was determined according to the ASTM E1820-11 Measurement of fracture toughness on reduced bodies for three-point bending. A minimum of four test specimens were used for each group of samples. Due to the fact that the tests do not meet the conditions of linear fracture mechanics, the calculation method via the J integral was used to evaluate the fracture toughness. In this case too, it is a transitional event and the sigmoidal function was again used to describe the dependence. The trend of the change in fracture toughness appears to be the opposite of the increase in the occupancy of grain boundaries by chromium carbides. The functional coefficients are shown in the graph in Figure 11; the coefficient of determination for the functional description is 99.32%, which again shows a high degree of description with the experimentally measured data.

## 4. Discussion

The precipitation of M_23_C_6_ carbides in HR3C steel has been extensively studied in the literature. Initial findings demonstrating the degradation of mechanical properties and the impact of carbide precipitation to the grain boundary cohesion were presented in [1]. Subsequently, a more comprehensive evaluation of the mechanical properties of exposed samples was conducted in [2], confirming the precipitation of chromium-rich M_23_C_6_ carbides along grain boundaries using SEM and TEM techniques. These carbides gradually coalesce into a continuous envelope along the grain boundaries. Further positive identification of M_23_C_6_ carbides has been reported in [8,9,10,14].

In this study, fracture surfaces of tested fracture toughness specimens were analyzed to assess fracture morphology. A rapid transition in fracture morphology was observed from ductile transgranular fracture in the base material to intergranular decohesion of grains.

A similar transition in fracture mode for HR3C steel has been reported in [3,5,6,8], although those studies focused on tensile and Charpy impact testing. These results collectively demonstrate a consistent influence of the degradation mechanism in HR3C steel. However, it is important to consider the different loading conditions, static (tensile testing), dynamic (Charpy impact testing), and methodology-dependent testing approaches outlined in [15] and the ASTM standard [13], which defines the procedure for determining fracture toughness.

From a fracture morphology perspective, austenitic HR3C steel exhibits, due to thermal exposure, a systematic transition from ductile transgranular fracture in the as-received state to intergranular fracture caused by grain boundary decohesion due to chromium carbide precipitation. This transition is evident under both static and dynamic loading conditions. In heterogeneous weld joints of HR3C/P92 steels, grain boundary fracture has also been documented [16,17]. The precipitation of M_23_C_6_ carbides not only weakens grain boundary cohesion but also significantly reduces mechanical properties. The influence of chemical composition on the precipitation of M_23_C_6_ in HR3C steel has been examined in [18], where it was shown that reduced chromium content decelerates mechanical degradation. In this case as well, the change in fracture mechanism was confirmed, consistent with findings from [3,5,8,9].

A key question remains regarding the influence of M_23_C_6_ precipitation on the energy at the crack tip during propagation and, consequently, on the fracture toughness of HR3C steel during prolonged thermal exposure.

**Figure 12:**
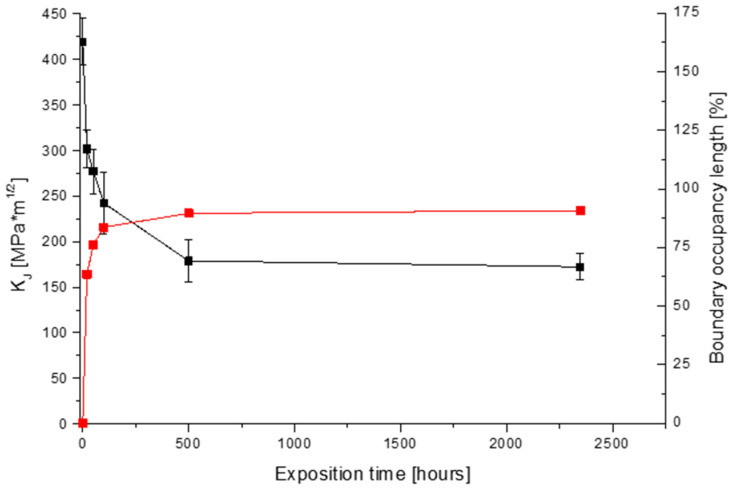
Change in fracture toughness and boundary occupancy length (BOL) with exposure time.

Figure 12 illustrates the dependence of fracture toughness degradation (black curve) on exposure time, showing a trend consistent with the decrease in impact energy reported in [8,9]. This is plotted alongside the inverse trend (red curve) of increasing precipitate-occupied grain boundary length (BOL). The inverse relationship between these two phenomena is evident. This correlation, along with the systematic changes in fracture surface morphology presented in this work and in [3,5,8,18], confirms the interdependence of these parameters. However, it is necessary to define a direct relationship between them. The use of the PML parameter seems to be a suitable element here because both precipitation processes and the decrease in fracture toughness induced by them are thermally activated events dependent on both the temperature and the time.

The application of the Larso–Miller Parameter (LMP) provides a means to generalize these dependencies across different exposure temperatures. This enables precise modeling of both fracture toughness degradation and BOL development using the following transition functions, Equation (5) (change in fracture toughness) and Equation (6) (the development of grain boundary occupation length).

(5) $$K_{J}=420.0059+\frac{269.1686}{1+e^{\frac{\left(LMP-18804.97\right)}{624.7425}}}$$

(6) $$BOL=91.5129-\frac{91.7047}{1+e^{\frac{\left(LMP-18217.46\right)}{450.9784}}}$$

In both cases, the coefficient of determination (R^2^) exceeds 99.3%, indicating an excellent fit to the experimental data.

**Figure 13:**
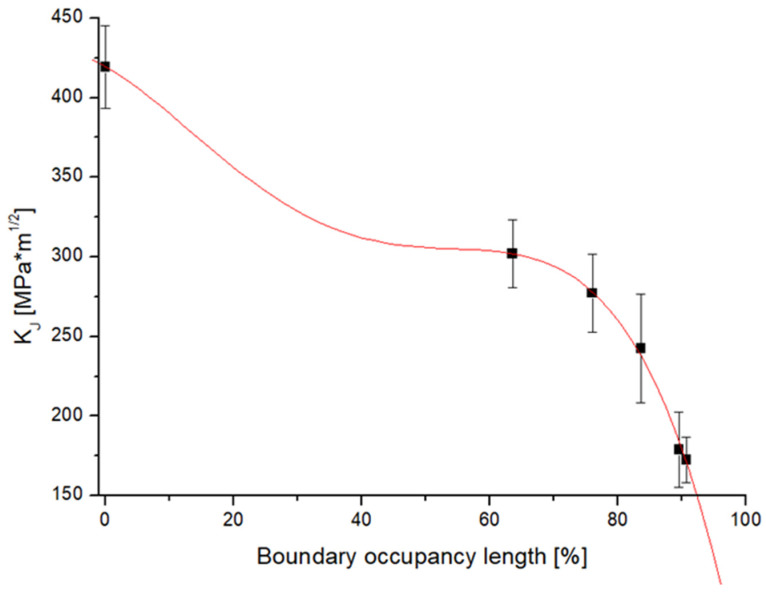
Regression of K_J_ to BOL.

A challenge in using LMP to describe fracture toughness lies in the non-isothermal operating conditions typical for power plants. Operational factors such as shutdowns, cooldowns, start-ups, and off-design states complicate accurate LMP estimation. These time–temperature variations, however, affect both M_23_C_6_ precipitation and mechanical degradation similarly, making BOL a more robust independent variable for describing material degradation. Therefore, a regression was developed to directly relate fracture toughness to BOL, as shown in Figure 13, with the following mathematical model (Equation (7)). This model achieves a coefficient of determination of R^2^ = 99.91%, providing a highly accurate representation of experimentally measured data.

(7) $$K_{J}=419.3984-2.2592BOL-0.0976{BOL}^{2}+0.00309{BOL}^{3}-2.28E^{-5}{BOL}^{4}$$

This regression allows for the estimation of fracture toughness in degraded HR3C steel, which is not readily available in the literature. References such as [2,8,9,18] provide only tensile or impact test data, with no direct correlation to fracture mechanics in components containing cracks. This model incorporates LMP as a connecting variable between K_J_ and BOL but uses BOL as the primary input for practical calculation. This enhances its applicability for assessing degradation through structural changes induced by thermal exposure. The model may be less accurate for BOL values below 50%, but such low degradation levels occur within only a few operational hours, as discussed in [2,8,9,18]. Therefore, some inaccuracy in this range is acceptable. In real-world power plant operations, materials typically reach and exceed BOL > 50%, which defines the practically relevant domain of this model. In practice, if the power plant is operated at 610 °C, the time required to reach BOL 50% will be approximately 300 operating hours, i.e., 12.5 days. This confirms that the inaccuracy for values below BOL 50% is indeed negligible.

## 5. Conclusions

As part of the operation and degradation characteristics of HR3C austenitic heat-resistant steel in energy blocks caused by its exposure to elevated temperature, it is necessary to consider the presence, development and propagation of cracks, caused by either production or degradation of the material. The general diagnosis of the presence and characterization of the size of cracks present in the material can be based on non-destructive methods of testing the material. However, this value alone does not provide enough information for the safe operation of the power block. For calculations of the critical size of the defect, it is necessary to know the fracture toughness of the material in the degraded state, which is not generally available for the HR3C material.

The literature [19,20] describes a method for determining fracture toughness for the Cr-Mo-V and high-strength austenitic steel material group; unfortunately, this method is not applicable in practice. A significantly wider application option for determining fracture toughness is to take surface structural replicas and determine the fracture toughness based on the occupancy of the boundaries. This will make it possible to comment on cracks in the system and determine the residual life.

The description, or the possibility of determining the fracture toughness for a value corresponding to up to 90,000 operating hours at a temperature of 610 °C, is provided by a graph and equations (Figure 13, Equation (7)). It is therefore a tool for determining the fracture toughness value on the basis of microstructural indicators (the boundary occupancy length) and thus obtaining input data for calculation models and determining the limit size of the defect. This is a critical rank for the safe operation of USC power plants with cracks presented in superheaters and reheaters.

The main contribution of this paper is to establish a relationship between fracture toughness and the state of material degradation covering most of the service life of HR3C steel. This, when applied in practice, will provide a tool to determine the fracture toughness of degraded HR3C steel as an input to subsequent strength calculations.

## Figures and Tables

**Table 1:** Chemical composition of the experimental material used.

Chemical Element	C	Si	Mn	P	S	Cr	Ni	Nb	N	Fe
wt.%	0.06	0.41	1.19	0.016	<0.001	24.9	19.9	0.44	0.26	bal.

## Data Availability

The original contributions presented in this study are included in the article. Further inquiries can be directed to the corresponding author.

## Abbreviations

The following abbreviations are used in this manuscript:

J	The value of the J integral
K_i_	Provisional value of fracture toughness
v	Poisson’s number
E	Yang’s modulus of elasticity
A_pl_	Plastic component of energy under the curve
B, B_N_, a, W, b_0_	Geometric parameters of the test body
α	Coefficient = 1
F_i_	Strength of the test endpoint
S	Distance of supports
K_JC_	Fracture toughness
